# Ontogeny of color development in two green–brown polymorphic grasshopper species

**DOI:** 10.1002/ece3.10712

**Published:** 2023-11-02

**Authors:** Mahendra Varma, Gabe Winter, Hannah M. Rowland, Holger Schielzeth

**Affiliations:** ^1^ Population Ecology Group, Institute of Ecology and Evolution Friedrich Schiller University Jena Jena Germany; ^2^ Max Planck Institute for Chemical Ecology Jena Germany

**Keywords:** color polymorphism, coloration, developmental plasticity, green–brown polymorphism, visual modeling, visual perception

## Abstract

Many insects, including several orthopterans, undergo dramatic changes in body coloration during ontogeny. This variation is particularly intriguing in gomphocerine grasshoppers, where the green and brown morphs appear to be genetically determined (Schielzeth & Dieker, 2020, *BMC Evolutionary Biology*, 20, 63; Winter et al., 2021, *Heredity*, 127, 66). A better understanding of how these color morphs develop during ontogeny can provide valuable insights into the evolution and ecology of such a widespread color polymorphism. Here, we focus on the color development of two green–brown polymorphic species, the club‐legged grasshopper *Gomphocerus sibiricus* and the steppe grasshopper *Chorthippus dorsatus*. By following the color development of individuals from hatching to adulthood, we found that color morph differences begin to develop during the second nymphal stage, are clearly defined by the third nymphal stage, and remain stable throughout the life of an individual. Interestingly, we also observed that shed skins of late nymphal stages are identifiable by color morphs based on their yellowish coloration, rather than the green that marks green body parts. Furthermore, by assessing how these colors are perceived by different visual systems, we found that certain potential predators can chromatically discriminate between morphs, while others may not. These results suggest that the putative genes controlling color morph are active during the early stages of ontogeny, and that green color is likely composed of two components, one present in the cuticle and one not. In addition, the effectiveness of camouflage appears to vary depending on the specific predator involved.

## Graphical abstract

Ontogenetic development of two species of gomphocerine grasshoppers. Color morph differences develop early and remain stable for the rest of the insects life.

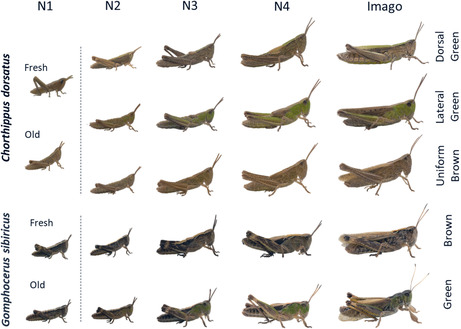

## 1 INTRODUCTION

Color polymorphism refers to the local coexistence of multiple discrete color phenotypes within a population, independent of sex and age (Ford, 1945; Huxley, 1955; White & Kemp, 2016). This phenomenon has sparked much theoretical and empirical interest in evolutionary biology (Svensson, 2017). Color polymorphisms are widespread across several taxonomic groups, including birds, mollusks, spiders, fish, mammals, and numerous insect orders (Bond, 2007; Darwin, 1859; Hoffman & Blouin, 2000; Mundy, 2005; Oxford & Gillespie, 1998; Whiteley et al., 1997). The study of color polymorphisms has played a central role in understanding how intraspecific diversity is generated and maintained (Brien et al., 2022; McKinnon & Pierotti, 2010). The question of why alternative color morphs coexist within a population, rather than one morph being fixed by natural selection or genetic drift, continues to be investigated (Endler, 1978; Mallet & Joron, 1999). Equal fitness of color morphs alone is not sufficient to prevent the loss of color variants in a population through genetic drift. Therefore, some form of balancing selection is usually required for their long‐term maintenance (Wellenreuther, 2017). Balancing selection can result from temporally or spatially heterogeneous selection, including frequency‐dependent predation (Endler et al., 1988; Ford, 1966; Madsen et al., 2022), and can involve physiological trade‐offs, such as between crypsis and thermoregulation (Hegna et al., 2013).

Orthopteran insects, which include crickets, bush crickets, and grasshoppers, provide a particularly striking example of color polymorphisms shared by several species (Dearn, 1990; Rowell, 1972). One such example is the green–brown polymorphism found in approximately 30% of all European orthopterans (Schielzeth, 2020) and 45% of East African acridid grasshoppers (Rowell, 1972). This green–brown polymorphism occurs in both Orthoptera suborders, Ensifera and Caelifera, which diverged ~355 million years ago (Mya) (Song et al., 2020). Even some other orders of polyneopteran insects, which diverged from Orthoptera about ~380 Mya (Misof et al., 2014; Song et al., 2020), show an equivalent green–brown polymorphism (Roth et al., 2014). Furthermore, the green–brown polymorphism in Orthoptera is geographically widespread, with a particularly high prevalence in grasslands (Schielzeth, 2020).

The striking green coloration observed in Orthoptera is thought to result from the interaction between blue bile pigments, such as biliverdin, and yellow carotenoid pigments, resulting in subtractive color mixing (Fuzeau‐Braesch, 1972; Okay, 1945, 1951). Thus, the production of green coloration requires the synthesis of blue bile pigments in epidermal cells (Fuzeau‐Braesch, 1972; Shamim et al., 2014), followed by its deposition in the integument. This process probably also involves the incorporation of yellow carotenoid pigments into the cuticle or epidermis. However, it remains unclear whether yellow carotenoids are also present in brown individuals. Even if both components are involved, the presence or absence of the blue component (bile pigment protein) may decisively determine whether the coloration appears green or not (Okay, 1953).

In Orthoptera, some species of gomphocerine grasshoppers (Caelifera, Acrididae), the green–brown polymorphism appears to have a simple genetic basis, with few loci controlling the color morphs and green alleles dominating over brown alleles (Schielzeth & Dieker, 2020; Winter et al., 2021). In other species, the development of green–brown phenotypes is triggered by environmental factors. High humidity favors the development of green morphs, whereas high temperatures and high population density favor brown morphs (Tanaka, 2004; Tanaka et al., 2012). Such changes between brown and green are usually associated with the molting process during the transition between nymphal stages. However, not all Orthoptera species respond to environmental cues. For example, in the cone‐headed grasshopper *Conocephalus maculatus* (an Ensiferan), the development of green or brown imagoes from green nymphs depends on parental morphs rather than environmental factors such as temperature, humidity, or substrate color (Oda & Ishii, 1998, 2001). Similarly, gomphocerine grasshopper nymphs do not appear to change color between green and brown morphs (Valverde & Schielzeth, 2015; Winter et al., 2021).

Many orthopterans also exhibit pattern polymorphism, which often involves differences between their dorsal and lateral sides and may include contrasts in the presence or absence of green coloration in different body parts (Rubtzov, 1935; Uvarov, 1966). These complex patterns may contribute to crypsis by disrupting the shape of the animal and may be subject to directional or frequency‐dependent selection (Cuthill et al., 2005; Madsen et al., 2022). In many gomphocerine grasshoppers, a pied morph typically lacks green coloration (Schielzeth & Dieker, 2020). However, in contrast to the uniform brown morph, the pied morph displays a distinctive black‐and‐white transverse pattern across the head and pronotum (Dieker et al., 2018).

Several orthopteran species also vary considerably in darkness, ranging from almost black to very light gray. This variation in darkness is believed to be caused by dark ommochromes and/or melanins, which tend to accumulate over the lifetime of an individual (Fuzeau‐Braesch, 1972; Valverde & Schielzeth, 2015). Changes in darkness in grasshoppers in response to environmental conditions are known as the ommochrome response (Rowell, 1970). Many orthopterans also show developmental plasticity in coloration, with individuals exhibiting shades of reddish, pink, yellow, orange, and purple (Peralta‐Rincon et al., 2017). These color variants are sometimes influenced by the environment, allowing individuals to plastically adapt to their local habitat. This adaptive process is known as the homochrome response (Rowell, 1970). The ommochrome and homochrome responses are gradually variable, in contrast to the green–brown polymorphism, which is largely discrete.

Currently, our understanding of the ontogeny of color development in grasshoppers remains limited. In this study, we aim to fill this gap by analyzing the color development in two gomphocerine species: the club‐legged grasshopper *Gomphocerus sibiricus* and the steppe grasshopper *Chorthippus dorsatus*. In gomphocerine grasshoppers, green–brown and pattern polymorphisms are commonly observed in both sexes, including the two species in our study. These species are paradigmatic for color polymorphic grasshoppers with genetically controlled color morphs (Schielzeth & Dieker, 2020; Winter et al., 2021). We followed individuals through their life stages to document the development of color phenotypes. In addition, we examined shed skin for differences between color morphs to assess whether color patches are reformed after each molt or if color persists in layers beneath the cuticle. Finally, we measured visual color with spectrometric measurements and used visual modeling to assess the ability of conspecifics and potential predators to discriminate between color morphs.

## 2 MATERIALS AND METHODS

### 2.1 Study area and subjects

We followed the individual development of color morphs in laboratory‐reared offspring of the club‐legged grasshopper *Gomphocerus sibiricus* and the steppe grasshopper *Chorthippus dorsatus*. Parental individuals were captured in the field in the summer of 2019 (*Gomphocerus sibiricus* in the French Alps, 45°4.5′ N, 6°25′ E, *Chorthippus dorsatus* in east‐central Germany, 50°56.5′ N, 11°36′ E) and mated in the laboratory. Eggs were collected and hibernated in standard refrigerators at 4–8°C. Eggs hatched in March 2020 after approximately 10–14 days at room temperature. All offspring were transferred to cages with ad libitum access to freshly cut grass placed in small water‐filled vials. Tubes of water with a cotton plug were provided for moisture.

There are three distinct morphs of *sibiricus*: green, brown and pied. Both brown and pied individuals lack green coloration and can therefore be classified as brown sensu *lato* (Dieker et al., 2018). In *dorsatus*, there are four color morphs: uniform brown, uniform green, lateral green, and dorsal green (Winter et al., 2021). The lateral and dorsal green morphs show a clear difference between the dorsal and lateral sides, with green restricted to one of these areas and the other part being brown. A total of 62 individuals of *sibiricus* (22 green, 16 brown, eight pied, and 16 unmorphed) and 59 individuals of *dorsatus* (24 uniform brown, 19 dorsal green, 10 lateral green, and six unmorphed) were used in this study. Ten nymphs of each species were housed separately in individual cages to follow their color development through ontogeny. The morph type and sex of each individual were unknown at the time of transfer to the cages. The remaining individuals were raised in groups of three. Individually housed grasshoppers that died were replaced with others from the pool of group‐housed individuals.

### 2.2 Photography

We aimed to document the color development of 10 individuals of each species from the first nymphal stage to adulthood. As some individuals had to be replaced, the total number of individuals for which at least part of the development was documented was 36 *sibiricus* and 25 *dorsatus*. Individuals were photographed in profile (lateral views) every 3–4 days on a homogeneous gray background using a DSLR camera (Canon EOS D7) with a macro lens (Sigma 150 mm Apo Makro DG HSM) and a ring light for illumination. The shed skins from the imaginal molt were collected and photographed on the same gray background. Images were captured in raw format and then corrected for the white balance and exposure across all images (using the invariable background color as a reference) using Adobe Lightroom Classic 11.2. We also photographed an additional 26 *sibiricus* and 34 *dorsatus* group‐housed imagoes under the same standardized conditions.

In addition, we performed color measurement analysis of standardized images from the lateral side of the head for 62 *sibiricus* individuals (238 images) and 59 *dorsatus* individuals (276 images). Using ImageJ 1.53p (Schneider et al., 2012), we measured the red, green, and blue (RGB) values of the images. Using the polygon selection tool in ImageJ, two random areas were selected: one on the lateral lobes of the pronotum and another on the lateral side of the head. The mean RGB value of these selected areas was measured. Since image exposure was manually adjusted, we measured only chromatic and not luminance differences. For all imagoes and late instar nymphs, morph identities were easily assigned (no color morph change was detected in our study). However, for some early instar nymphs that died prematurely, no color morph could be confidently assigned. The RGB values were then used to create ternary plots using the R package *ternary* 2.1.3 (Smith, 2017). Despite the limitations of using RGB color analysis from uncalibrated photographs (Stevens et al., 2007), our analyses allowed us to quantitatively measure the basic color metrics of individual grasshoppers and provide information on at what stage of development the green and brown colors begin to differentiate. However, these data do not allow us to determine whether these color differences would be perceived by different visual systems.

### 2.3 Reflectance measurements

We measured reflectance in adults using a handheld spectrophotometer (Avantes AvaSpec‐ULS2048) with a halogen deuterium light source (Avantes, Ava‐Light‐D(H)‐S). Individuals were illuminated and measured perpendicular to the surface. We set the integration time to 100 ms and the spectrometer automatically averaged five readings for a single measurement. We measured reflectance on the lateral lobes and dorsal side of the pronotum of 38 individuals of *sibiricus* (13 green females, nine green males, nine brown females, and seven brown males) and 34 individuals of *dorsatus* (two brown females, 10 brown males, seven dorsal green females, seven dorsal green males, four lateral green females, and four lateral green males). The spectrophotometer was calibrated with a commercial white standard (Avantes WS‐2) before each patch was measured. We measured each patch in five locations per individual to cover the entire area. We averaged the five measurements per patch per individual for analysis and removed noise using functions implemented in the R package *pavo* 2.4.0 (Maia et al., 2019). This resulted in 76 averaged reflectance spectra (38 individuals * 2 patches) for *sibiricus* and 68 (34 individuals * 2 patches) for *dorsatus*. In the raw spectrometric readings, a sharp peak between 653.5 and 660.5 nm was an apparent artifact of the instrument and was removed by averaging in the ranges 650–653 nm and 661–664 nm (Heinze et al., 2022). Note that this correction affected only a very small region of the entire spectrum.

### 2.4 Visual modeling

Visual models were used to determine whether specific predators could discriminate between color morphs. For the visual modeling analysis, we used spectrometric measurements and the R package *pavo*. The wavelength range considered for visual modeling was 300–700 nm. To ensure that only non‐negative values were retained, we used the *addmin* option in *pavo*, which adjusts negative values by adding an offset. No other manipulations were made. All analyses were performed in R 4.1.1 (R Core Team, 2018).

Our study considered visual models for both trichromatic and tetrachromatic species, each representing potential grasshopper predators (or their relatives). Trichromatic species possess visual pigments that allow them to perceive information in three different wavelength ranges: long, medium, and short. In contrast, tetrachromatic species can perceive ultraviolet and violet wavelengths in addition to the three primary cone types. We considered three trichromatic species in our modeling, a lizard (*Ctenophorus ornatus*; λ_max_ at 571 nm (long‐wavelength sensitive, LWS), 493 nm (medium‐wavelength sensitive, MWS), and 440 nm (short‐wavelength sensitive, SWS) (Barbour et al., 2002)), a jumping spider (*Habronattus pyrrithrix*; λ_max_ at 626 nm (LWS), 530 nm (MWS), and 377 nm (UV‐sensitive) (Zurek et al., 2015)), and the honey bee (*Apis mellifera*; λ_max_ = 544 nm (LWS), 436 nm (MWS), and 344 nm (SWS) (Menzel & Backhaus, 1991)). For tetrachromatic species, we considered the housefly (*Musca domestica*; λ_max_ at 520 nm (LWS), 490 nm (MWS), 420 nm (SWS), and 360 nm (UV sensitive) (Hardie & Kirschfeld, 1983)), the European starling (*Sturnus vulgaris*; λ_max_ at 563 nm (LWS), 504 nm (MWS), 449 nm (SWS), and 362 nm (UV sensitive) (Hart et al., 1998)), and the peafowl (*Pavo cristatus*; λ_max_ at 605 nm (LWS), 537 nm (MWS), 477 nm (SWS), and 432 nm (violet sensitive) (Hart, 2002)). Lizards, spiders, and birds are important predators of grasshoppers (Ingrisch & Köhler, 1998), and the two insects were chosen to represent predatory and parasitoid wasps and flies that prey on and infect grasshoppers (Ingrisch & Köhler, 1998). Honey bee's peak cone‐catch sensitivities are similar to those of the migratory locust *Locusta migratoria* (Briscoe & Chittka, 2001), making it a proxy for grasshopper vision. To our knowledge, full sensitivity curves for grasshopper cones are not currently available.

In our visual modeling analysis, models were implemented with flat, full‐spectral illumination, and a wavelength‐independent background effect on color perception, using the *ideal* option in *pavo*. To compute noise‐weighted chromatic and achromatic visual distances between morphs, we adopted the receptor noise model proposed by Vorobyev et al. (Vorobyev et al., 1998). This model is based on the relative photoreceptor densities of six animals, which serve as proxies for potential predictors. We fitted this model using the *coldist* function in *pavo*, which quantifies both chromatic contrast (ΔS) and achromatic contrast (ΔL). Smaller values of ΔS and ΔL indicate greater similarity in coloration from the predator's perspective, while larger values indicate more significant differences. In our analysis, when both ΔS and ΔL exceed 3, we conclude that the morphs are discriminable by the predator. Furthermore, for achromatic distances, we used the starling double‐cone model for starling, the chicken double‐cone model for peafowl, the house fly R1‐6 photoreceptor model for house fly, and the summed response of all photoreceptors for all other species. Additionally, we used homogeneous transmission (*ideal* option in *pavo*) and noise proportional to the Weber fraction (*neural* option in *pavo*) to model visual distance.

## 3 RESULTS

### 3.1 Color development

**FIGURE 1:**
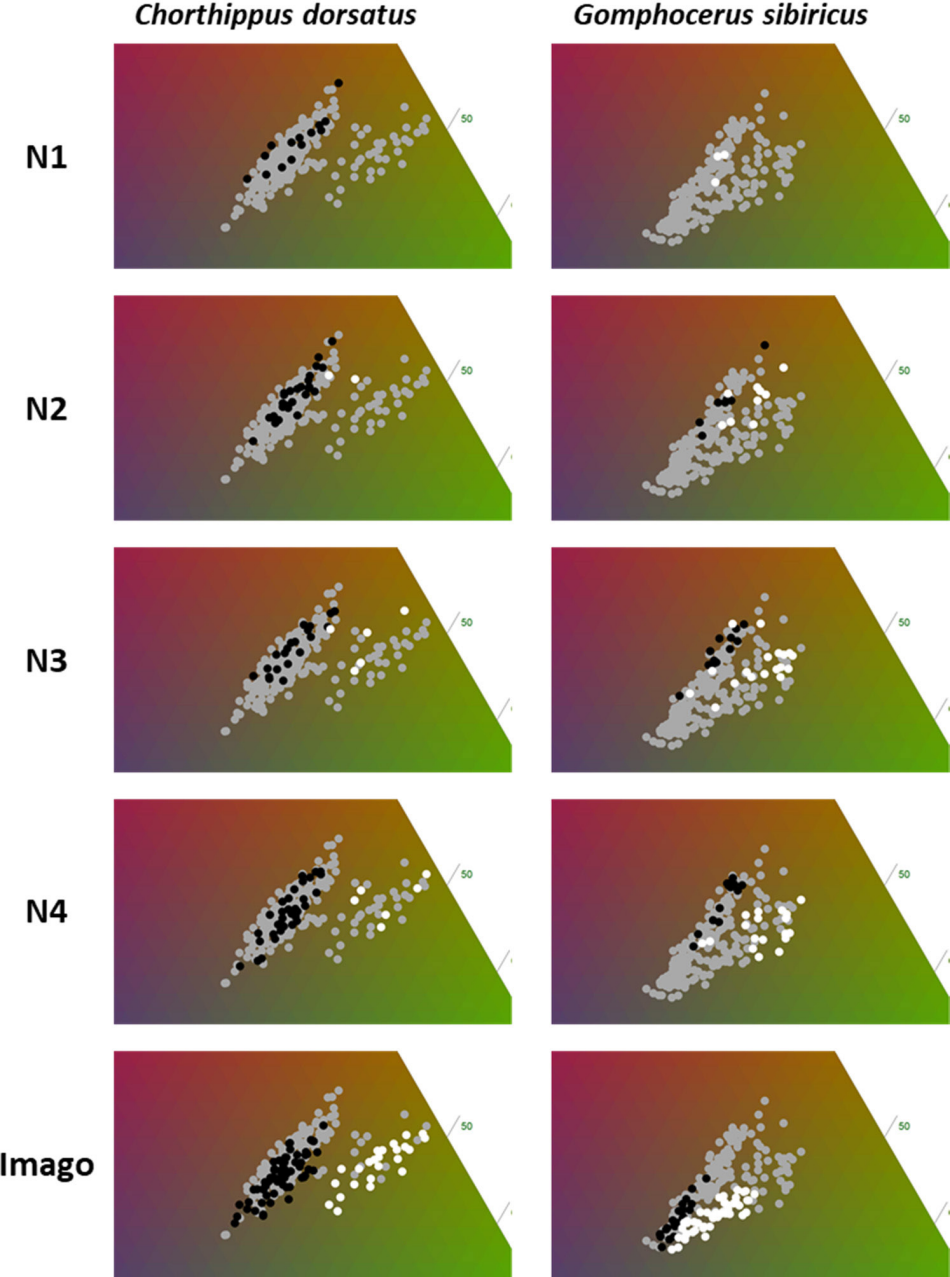
Chromatic development of *Gomphocerus sibiricus* and *Chorthippus dorsatus*. Plots show the distribution of red, green, and blue (RGB) values extracted from standardized images of (lateral) head coloration. Values are plotted on a ternary plot of which the relevant section is shown. Gray dots show the distribution of all measurements (of a given species) and serve as a reference across all plots. Black dots show brown individuals (including pied morphs for *sibiricus* and dorsal green for *dorsatus*) while white dots show green individuals (including lateral green for *dorsatus*).

**FIGURE 2:**
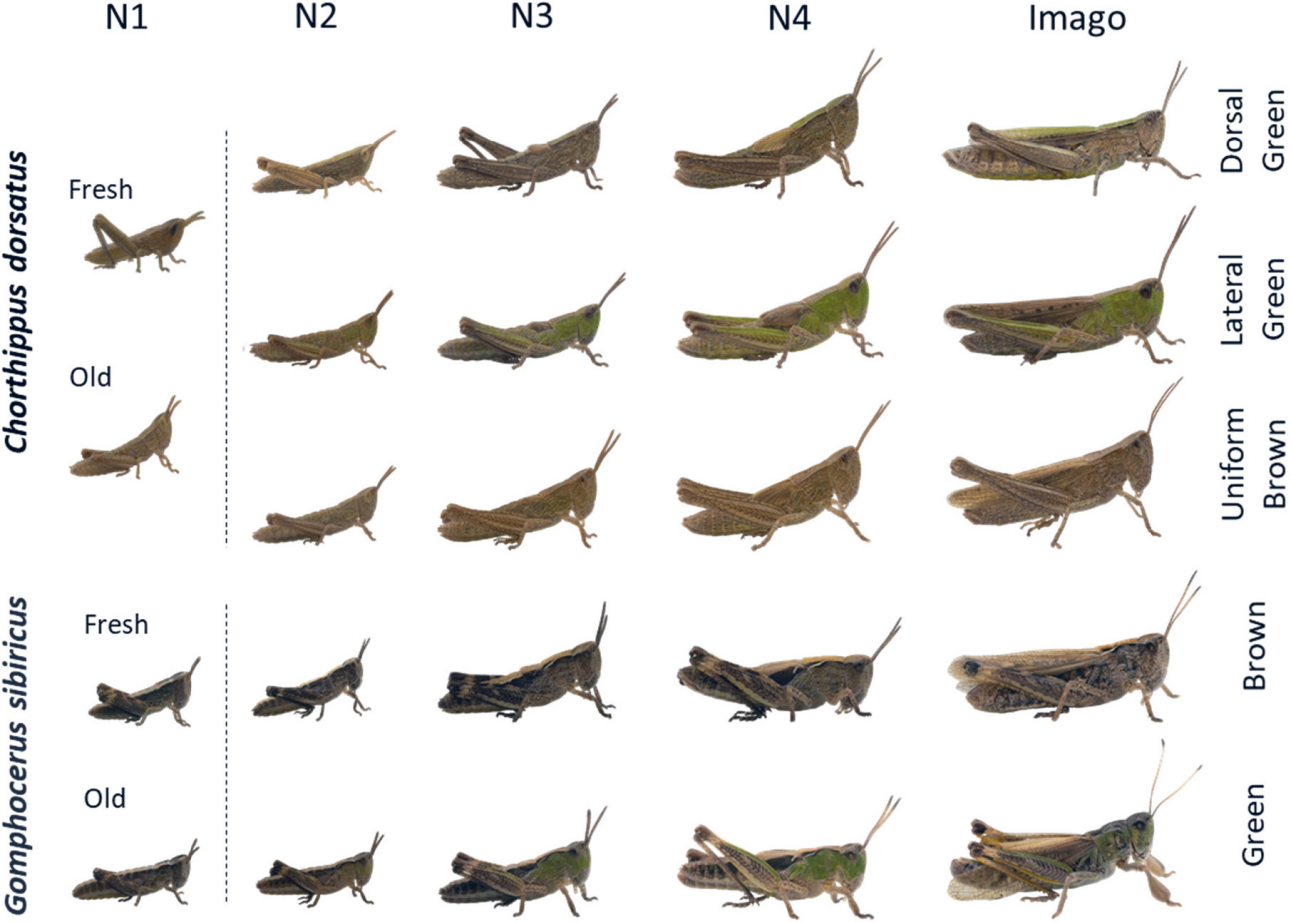
Ontogenetic development of two morphs of *Gomphocerus sibiricus* and three of *Chorthippus dorsatus*. The individuals with the most complete series of pictures from the second nymphal to the imaginal stage were selected for illustration. The individuals from the first nymphal stage (separated by dotted lines) belong to different individuals and are not part of this set. Color morphs remain stable once expressed in nymphal stages 2 or 3 throughout development.

The two species completed their ontogenetic development of four nymphal stages after 21–37 days. During the first nymphal stage, the color morphs appear indistinguishable, as can be seen from the co‐clustering in RGB space (Figures 1 and 2). Newly hatched individuals are pale at hatching (when the cuticle is still soft), but become very dark (almost black) within a few hours, particularly in *sibiricus*. The differences between the color morphs became more evident from the second nymphal stage (N2) onwards. In general, the green color intensified within a few days after each molt, so that the color morph of old N2 is clearly identifiable upon close inspection, while young N2 are still ambiguous. When individuals were in their third nymphal stage (N3), the color morphs of both species were distinguishable based on their RGB profiles (Figures 1 and 2).

Once morph differences were developed in the individuals, the color was stable throughout life and we observed no further color morph changes. The green color was most pronounced on the head and pronotum and extended to the abdomen, legs, and wings. Similarly, the dorsal–lateral color polymorphism in the *dorsatus* was visible from the N2 stage and became more pronounced in the N3 stage. Morph‐specific coloration was evident somewhat earlier during development (N2) in *sibiricus* than in *dorsatus* (Figure 1). Interestingly, in both species, but especially in *sibiricus*, the imagoes become clearly blue shifted (Figure 1).

**FIGURE 3:**
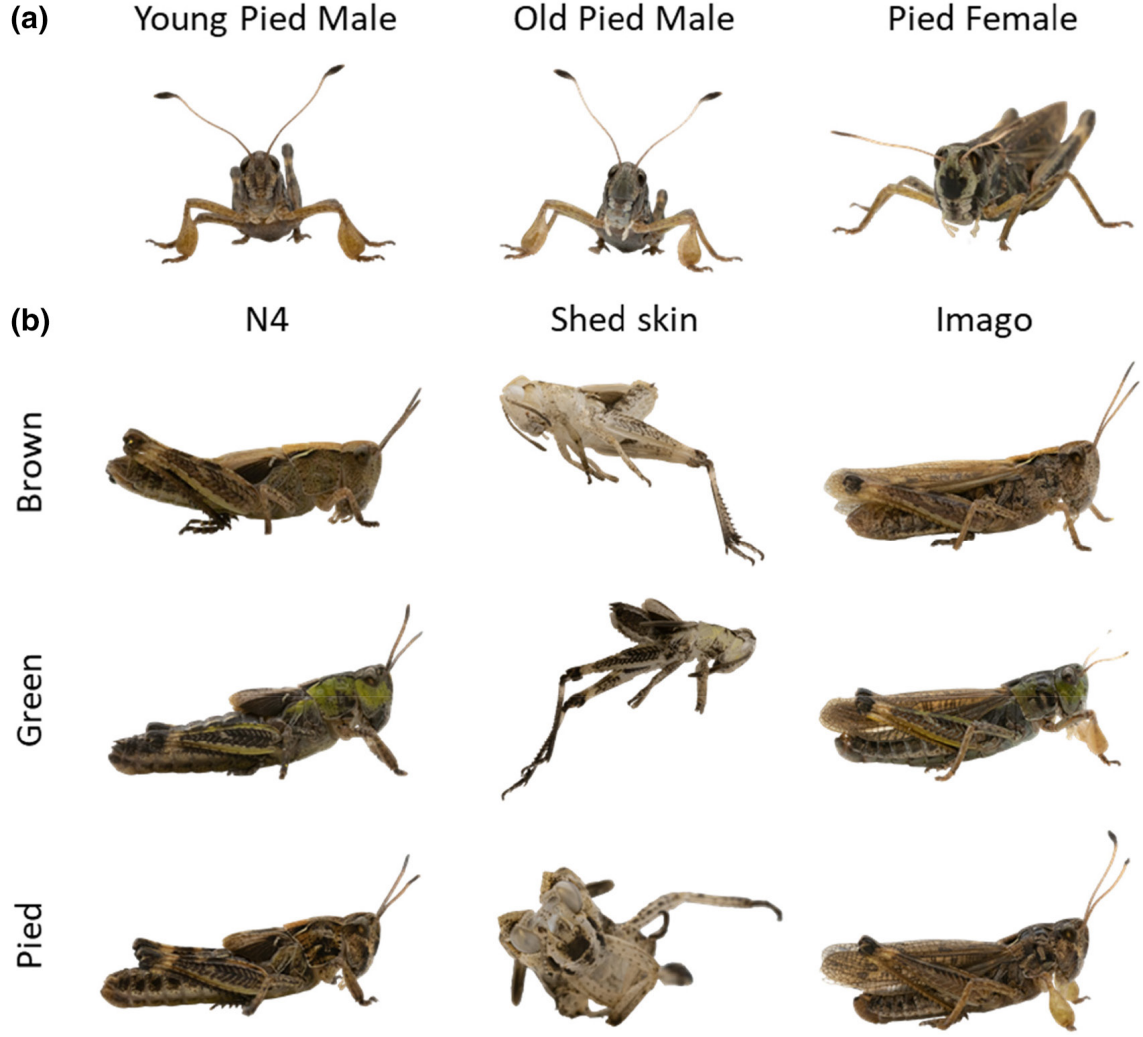
(a) Post‐imaginal morph development of pied *Gomphocerus sibiricus*. A bold black patch is a characteristic of the pied morph. The patch is present in both sexes in stage N4, but it fades in males within a few days after the final molt, while it remains marked in females. (b) Shed skins from the fourth nymphal stage of *Gomphocerus sibiricus*. Skins show the distinct imprint of black pigments irrespective of the color of the individuals. Apart from black traces, a tinge of yellow color is present on the skin of green individuals in contrast to brown/pied individuals.

Males and females of club‐legged grasshoppers show a distinct pied color morph during the late nymphal stages. The pied color is characterized by a diffuse white transverse lateral band across the head and the pronotum, and a bold black patch on the front of the head (which may also be missing in some individuals) (Dieker et al., 2018). Interestingly, this pattern persists into adulthood in females and is marked throughout their adult lives (Figure 3a). In males, however, the pied pattern completely blurs within a few days after the imaginal molt. This makes pied adult males almost indistinguishable from brown males about 3–4 days after the final molt (Figure 3a). The only remaining difference is a darker front, which is retained in pied males.

### 3.2 Color imprints on shed skin

Shed skins are predominantly pale and transparent. We focus here on the final skin that was left after the imaginal molt. There was no green color in the shed skin, but the skins of brown and green individuals were distinguishable by a dispersed faint yellow hue in green individuals. In contrast to the absence of green, black patches were very prominent in the shed skin. Skins from pied individuals, for example, are easily recognizable by the bold black front patch that is clearly visible on the shed skin (Figure 3b).

### 3.3 Spectrometric measurement analysis

**FIGURE 4:**
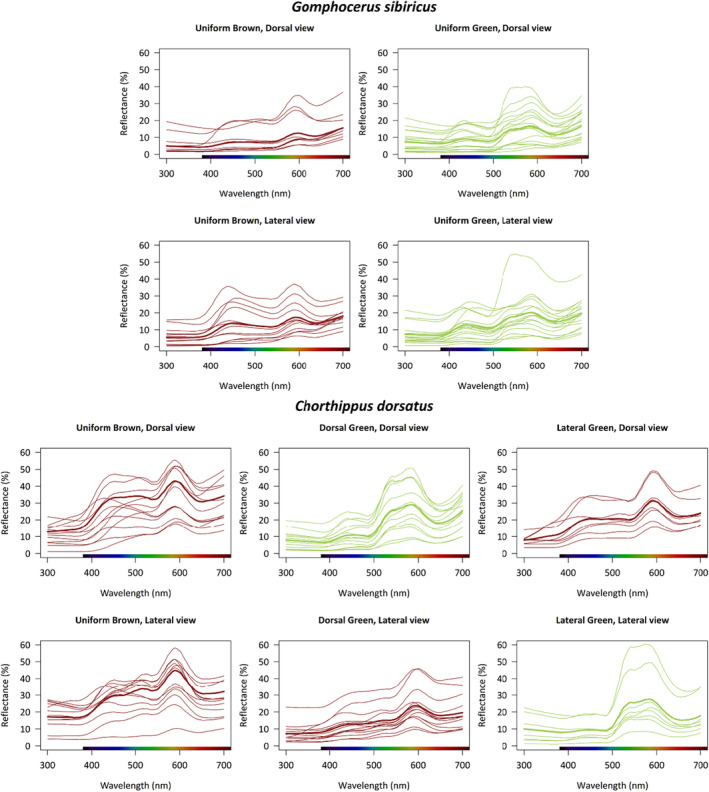
Reflectance pattern in club‐legged grasshopper *Gomphocerus sibiricus* and the steppe grasshopper *Chorthippus dorsatus*. Reflectance was measured on the lateral and dorsal sides of the pronotum in two morphs of *sibiricus* (pied here pooled with brown) and three morphs of *dorsatus*. Thin lines show the reflectance of single individuals (averaged across five measurements), while thick lines show reflectance curves averaged across individuals.

We measured reflectance on both the lateral and dorsal sides of adult individuals. The reflectance profiles showed significant differences between morphs in both species (Figure 4). Specifically, brown morphs have higher reflectance at shorter wavelengths (violet–blue) compared to green morphs. The green morphs are characterized by two peaks at 520 and 580 nm, whereas the brown morphs are characterized by a single peak at 590 nm in the red part of the spectrum (Figure 4). The two species, *sibiricus* and *dorsatus*, showed similar reflectance patterns in their green and brown morphs (Figure 4).

Dorsal green morphs showed a spectral profile similar to green on the dorsal side and brown on the lateral side. Lateral green morphs showed the reverse pattern, with spectral profiles similar to brown on the dorsal side and green on the lateral side (Figure 4). These similarities are consistent with our classification of color morphs (green patches being alike and brown patches being alike). Overall, *sibiricus* showed substantially more variability between independent measurements. This reflects their patchier patterns compared to *dorsatus*.

### 3.4 Visual modeling

**FIGURE 5:**
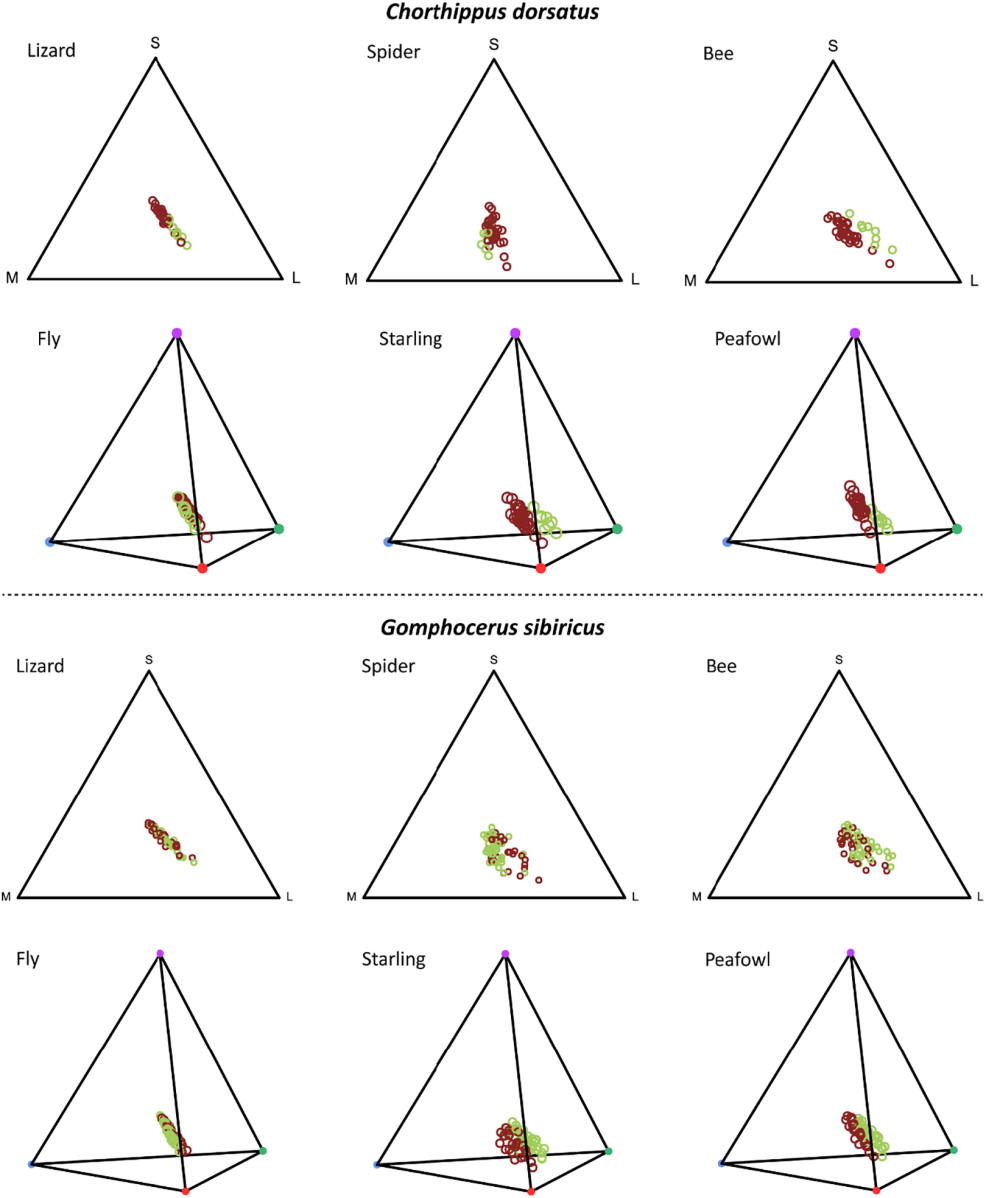
Visual models of reflectance spectra of *Chorthippus dorsatus* and *Gomphocerus sibiricus* as perceived by representatives of potential predators. Each circle represents one grasshopper individual with a brown circle for brown individuals and a green circle for green individuals.

Despite the marked differences in the reflectance profiles between color morphs, visual modeling suggests that lizards and flies cannot chromatically discriminate between morphs (Table 1). Birds (peafowl and starling), however, are predicted to discriminate the green and brown colors of grasshoppers (Figure 5). Nevertheless, all predator species are predicted to discriminate between color morphs when luminance is considered. The visual modeling results were more distinct with respect to morph differences in *dorsatus*, probably reflecting the more heterogeneous patterning of *sibiricus*.

**TABLE 1:** Comparisons of chromatic (ΔS) and achromatic (ΔL) distances between color patches of *Gomphocerus sibiricus* and *Chorthippus dorsatus* and as modeled by the visual models for six species representing potential predators.

	Trichromatic species						Tetrachromatic species					
	Lizard		Spider		Bee		Fly		Starling		Peafowl	
	ΔS	ΔL	ΔS	ΔL	ΔS	ΔL	ΔS	ΔL	ΔS	ΔL	ΔS	ΔL
*Gomphocerus sibiricus*												
Brown vs. green body parts												
Green vs. brown morphs (dorsal view)	0.78	**3.82**	1.74	**3.58**	1.03	**4.14**	1.3	**3.9**	2.18	**4.5**	2.23	**4.38**
Green vs. brown morphs (lateral view)	1.31	0.32	0.64	0.06	1.4	0	1.35	0.33	1.88	0.76	1.92	0.72
Brown vs. brown body parts												
Brown morphs (lateral vs. dorsal side)	1.3	**4.97**	1.21	**3.86**	2.1	**4.6**	1.43	**4.9**	2.04	**4.11**	1.94	**4.02**
Green vs. green body parts												
Green morphs (lateral vs. dorsal side)	0.74	0.84	0.51	0.33	1.5	0.45	1.04	0.67	1.25	0.38	0.97	0.36
*Chorthippus dorsatus*												
Brown vs. green body parts												
Brown vs. dorsal green morphs (dorsal view)	**3.32**	**4.76**	1.79	**3.87**	**3.07**	**4.66**	2.76	**5.2**	**3.73**	2.34	**3.76**	2.35
Brown vs. lateral green morphs (lateral view)	**3.56**	**7.56**	2.04	**6.69**	**3.32**	**7.59**	2.97	**8.02**	**4.08**	**4.88**	**3.96**	**4.9**
Lateral green vs. dorsal green morphs (dorsal view)	**3.48**	2.98	2.65	2.35	**3.46**	**3.05**	**3.02**	**3.41**	**3.81**	0.46	**3.72**	0.5
Lateral green vs. dorsal green morphs (lateral view)	2.11	0.19	1.83	0.3	2.61	0.68	2.18	0.31	2.99	1.61	2.85	1.51
Lateral green morphs (lateral vs. dorsal side)	**3.27**	**4.27**	1.49	**3.56**	**3.7**	**3.78**	2.96	**4.27**	**3.9**	1.96	**3.76**	2.03
Dorsal green morphs (lateral vs. dorsal side)	2.32	1.47	2.18	1.51	2.56	1.4	2.38	1.18	2.76	**3.11**	2.69	**3.04**
Brown vs. brown body parts												
Brown vs. lateral green morphs (dorsal view)	0.39	1.77	0.89	1.52	0.55	1.61	0.41	1.79	0.61	1.87	**0.64**	1.86
Brown vs. dorsal green morphs (lateral view)	1.51	**7.75**	2.41	**6.99**	1.51	**8.26**	1.23	**8.34**	2.34	**6.49**	2.14	**6.4**
Brown morphs (lateral vs. dorsal side)	0.47	1.52	1.99	1.61	2.08	2.2	1.55	1.96	1.49	1.04	0.56	1.01

*Note*: Delta values greater than 3 are shown in bold.

## 4 DISCUSSION

In this study, we document ontogenetic color development in two species of gomphocerine grasshoppers and analyze the spectral properties of color polymorphic body regions. We observed that during the first of four nymphal stages, individuals do not exhibit visible color polymorphism. It is in the second nymphal stage that distinct color morphs become discernible to the human eye. The color morph of third instar nymph and older is easily identifiable. Once developed, all individuals maintain their color morph throughout life. The green color is not visible in the shed skin, although the skin of green individuals has a yellowish tinge, suggesting that some component of the green color remains in the cuticle. In contrast, the marked black color patches of the pied morphs are clearly visible in the shed skin and are thus based, at least partly, on pigments deposited in the cuticle. Visual modeling of the spectrophotometric data shows that the green color patches of green individuals of both species are qualitatively similar, as are the green patches of the bicolor morphs of the steppe grasshoppers. Visual modeling also shows that only some potential predators can discriminate color morphs chromatically, but all selected species can discriminate color morph differences in luminance.

Our data have implications for how the color polymorphism is formed. In the two gomphocerine species studied here, the polymorphism has been shown to have a heritable basis and appears to be based on only a few loci with a dominance of a putative green allele over a brown allele (Schielzeth & Dieker, 2020; Winter et al., 2021). We did not observe changes in the color morph after its initial expression during ontogeny, confirming previous studies that have shown that green coloration has a genetic basis and that a single dominant allele could cause green coloration. However, our data suggest that the green coloration is not expressed in the first nymphal stage. Green body areas appear and become more intense during ontogeny. Despite individual variation, individuals can generally be assigned to their color morph during the second nymphal stage (with some remaining ambiguity in young N2) and with greater confidence during the third nymphal stage. Thus, the putative gene allele appears to be effective from the second nymphal stage onwards and then intensifies.

Grasshoppers undergo significant changes in their appearance during the nymphal stages, often becoming darker as they mature (Valverde & Schielzeth, 2015). Interestingly, we find that the green color also intensifies within the nymphal stages, particularly in the second stage and to a lesser extent in the third stage. However, it remains an open question whether the intensification of the green color within stages is caused by the continued deposition of pigments or by changes in the cuticle structure that make the pigments more visible. The green color does not appear to be present in the cuticle, so its primary location is most likely in the epidermal cells. The fact that green morphs can be identified by shed skin suggests that the green coloration is due to the presence of two components. The combination of yellow pigments, possibly carotenoids or variants of pheomelanin, interacting with blue pigments such as biliverdin likely produces the green appearance (Okay, 1945, 1951, 1953). Interestingly, we find a blue‐shifted color in imagoes, possibly indicating the presence of blue pigments. Future research is needed to test for the presence of blue biliverdin in the epidermis and some yellow component(s) in the epidermis and/or cuticle.

In contrast to the lack of green color, black patches, including those typical of pied individuals, are markedly present in the cuticle. The black coloration is probably due to (eu)melanins produced by a specialized group of cells called melanocytes. Individuals darken within nymphal stages, suggesting an accumulation of melanins in the subcuticular layers, while black pigments remain in the cuticle. This suggests that melanins are incorporated into the cuticle during the synthesis of the new exoskeleton prior to molting, while they continue to accumulate in the living epidermal layer after molting. This may explain the loss of the distinctive pied phenotype in club‐legged grasshopper males. While males are easily identified as pied morphs during the nymphal stages (as with green morphs, mostly from the second stage onward) and for a few days after final ecdysis, the pattern becomes completely blurred a few days into the imago stage, presumably due to the accumulation of melanins in the epidermis. The black pigmentation thus appears rather dynamic.

Spectral analyses show that the green colors are qualitatively similar between the two species studied here and a third species, the meadow grasshopper *Pseudochorthippus parallelus*, that has been analyzed previously (Heinze et al., 2022). Although the same green color could be produced by a variety of pigments (alone or in combination), these results are consistent with the hypothesis that the pigments—and possibly also the genetic pathway leading to them—are shared among gomphocerine grasshopper species. There are also interesting ecological implications if colors are shared among species. Most grasshopper habitats host multiple species. Although the club‐legged grasshopper and the steppe grasshopper do not typically occur in sympatry (at least in Europe), they both occur with the meadow grasshopper, which has a very similar green reflectance spectrum (Heinze et al., 2022). Although we do not document a cryptic value of green color here, an obvious hypothesis is that the green–brown polymorphism is maintained at least partly by improved crypsis of the green variant, possibly involving a trade‐off with thermoregulation (Köhler, 2006; Köhler & Schielzeth, 2020).

Even in the absence of morph‐differential crypsis, polymorphism could be maintained if predators develop search images and specialize on the most abundant morph in a given habitat (Bond, 2007). If color morphs are shared among species, selection might not act solely on the level of individual species but rather at the level of morphologically and behaviorally similar species, such as all sympatric gomphocerine species. However, to our knowledge, community‐level selection and its effects on the maintenance (or loss) of polymorphism have never been studied in grasshoppers.

Overall, our data show that color morphs are expressed quite early during ontogeny, that morph differences are stable, and that at least part of the green coloration is located in the epidermis. The pied morph, on the contrary, appears to be formed by pigments that are deposited in the cuticle and thus must be reformed with each molt. Despite this difference, the pied morph also appears to be ontogenetically stable. The similarity in green coloration among species not only tentatively suggests a shared genetic mechanism but also opens up the possibility of community‐level selection on grasshopper colors. Such avenues should be pursued in the future. We hope that our data will inform both biochemical analyses (e.g., differential gene expression analysis targeting appropriate stages and tissues) and advanced techniques such as Raman spectroscopy and mass spectroscopy (e.g., pigment identification) in future studies, as well as ecological studies on the selection pressures that maintain the green–brown polymorphism.

## AUTHOR CONTRIBUTIONS

**Mahendra Varma:** Conceptualization (equal); data curation (lead); formal analysis (lead); investigation (lead); methodology (lead); validation (lead); visualization (lead); writing – original draft (lead); writing – review and editing (lead). **Gabe Winter:** Formal analysis (supporting); funding acquisition (supporting); visualization (supporting); writing – review and editing (supporting). **Hannah M. Rowland:** Methodology (supporting); validation (supporting); visualization (supporting); writing – review and editing (supporting). **Holger Schielzeth:** Conceptualization (lead); formal analysis (equal); funding acquisition (lead); investigation (lead); supervision (lead); visualization (supporting); writing – original draft (supporting); writing – review and editing (supporting).

## Data Availability

Data available from the Dryad Digital Repository: https://doi.org/10.5061/dryad.wdbrv15vc
